# From biomechanics to sport psychology: the current oscillatory approach

**DOI:** 10.3389/fpsyg.2015.01642

**Published:** 2015-10-31

**Authors:** Guy Cheron

**Affiliations:** ^1^Laboratory of Neurophysiology and Movement Biomechanics, ULB Neuroscience Institut, Université Libre de BruxellesBrussels, Belgium; ^2^Laboratory of Electrophysiology, Université de Mons-HainautMons, Belgium

**Keywords:** movement, oscillation, sports, dynamics, tensegrity, strategies, sensorimotor control

Brain oscillations are, perhaps paradoxically, crucial for movement stability and high performance. Much of what is known about brain oscillations and their relation to movement, sensation and cognition has been established during the last three decades through an explosion of research ranging from *in vitro* (Draguhn et al., 1998; Fisahn et al., 1998) to *in vivo* (Cheron et al., 2015) and computing (Cannon et al., 2014) studies and reaching direct application to humans (Lebedev and Nicolelis, 2006; Schneider et al., 2010; Zarka et al., 2014). In this Grand Challenges monograph, my intent is to expound the idea that recording of multiple biological signals including brain oscillations during sport movements makes the most of currently available knowledge in neuroscience, technological advances and powerful analysis tools to promote excellence in sports performance. It is a scientific *voie royale* reinforcing the strong yet complex link between movement science and sport psychology.

## For the integration of movement and neuropsychological determinants

The dynamic nature of biological movements of both animals and humans has always fascinated human culture. However, artists (since prehistoric times) and scientists have been confronted with the difficulty of keeping the whole movement and its psychological meaning in one fixed picture due to the fugacity of individual movements. Despite the accumulation of scientific data in the field of movement science and related psychology, this challenge has remained incompletely met. Biological movements of humans and other animals remain commonly analyzed independently from their underlying neurophysiological mechanisms. In the last decades, major technological developments in the recording of 3D movement kinematics, kinetics and dynamics have contributed to bridge the gap between movement capture and the understanding of the internal control of movement by the brain. Yet, current knowledge remains sparse, unstable, and at times controversial, particularly in the niche of sport psychology, although it would be central to any progress. Whatever the sport movement considered, the actual performance and the related mental set are intrinsically linked and comprehensive understanding of this link is now indispensable for further performance optimization, not only *Citius, Altius, Fortius* (the Olympic motto, latin for “faster, higher, stronger”) and above all finer and more human.

## From muscle patterns to artificial dynamic neural network

Since the key observations of Aristotle about locomotion and then the first mechanical model of this seeming simple movement proposed by Borelli (1680) followed by Marey's first real-time kinematic representations as stick pictograms (1901), fine scientifically minded observers engaged onto the path of Movement Science questioned its most basic tenet: *the relations between muscles and movements* (Marey, 1901). This set the base for conventional movement analysis, integrating kinematics, kinetics and electromyography (EMG; Bengoetxea et al., 2014, 2015). Such a multimodal approach to movement has generated a wealth of data whose analysis requires the necessity to include the compliance of the musculoskeletal system (Gottlieb, 1996) and the redundancy problem (Neilson, 1993; Sporns and Edelman, 1993; Hayashibe and Shimoda, 2014).

Numerous models have thus taken tendon function into account (Winters et al., 1988; Zajac, 2002; McGowan et al., 2013), confirmed by ultrasound data about the musculoskeletal system in movement (Cronin and Lichtwark, 2013). The traditional view about force transmission considers that the force produced by the motor unit contractile element is uniquely transmitted to the bone via the tendon. This simplified assumption needs to be revisited by the integration of the connective fascia which binds muscles together and with other tissues assuming lateral transmission of force (Huijing and Jaspers, 2005; Higham and Biewener, 2011). Such force diffusion largely depends on the considered body segments (Gandevia, 2014). For example, there is no intermuscular force transmission between medial gastrocnemius and soleus in human (Tian et al., 2012). In contrast, non-linear force transmission results e.g., from active contraction of flexor digitorum profundus, which can generate no force at its finger insertion (Van Duinen and Gandevia, 2011). This calls for the necessity to record multiple EMG.

According to some authors, EMG patterns are a good reflection of the motor program used by the central nervous system for controlling movement (Gottlieb, 1993). However, for the tenants of the equilibrium point theory, EMG patterns are emergent and non-programmable properties of the system, which is controlled by a subtle combination of threshold muscle length λ of the implicated muscles (McIntyre and Bizzi, 1993; Feldman et al., 1998; Gribble et al., 1998; Feldman and Levin, 2009). Whatever the control variable of these signals, the EMG envelope signals have been shown to reasonably reflect the firing rate of motoneuronal pools including both central and afferent influences (Cheron and Godaux, 1986). In addition, the combination of EMG from multiple muscles may reveal the basic motor coordination dynamics of the gesture (Scholz and Kelso, 1990; Kelso, 1995). In this context, the utilization of dynamic recurrent neural network (DRNN), recognized as universal approximators of dynamical systems (Doya, 1993; Schäfer and Zimmermann, 2007) allows identification of the complex relationship between EMG signals and kinematics. This was made for fast upper limb figurative movements (Cheron et al., 1996), whole-body straightening (Draye et al., 2002), pointing ballistic movement (Cheron et al., 2007) and locomotion (Cheron et al., 2003, 2012; Hoellinger et al., 2013). This neuronal approach of movement science has also been applied to quantify maturational aspects of movement and to decipher strategic characteristics of elite sport performance, e.g., lunging in fencing champions (Cheron et al., 2011). In accordance with Bernstein's view (Bernstein, 1967), in both of these situations dynamic patterns emerge through exploration of available solutions to the redundancy problem, leading to selection of preferred movements. For example, in toddlers the rhythmic leg patterns progressively emerges through repeated cycles of action and perception ending in a planar covariation pattern (Cheron et al., 2001). This intersegmental coordination rule (Borghese et al., 1996; Lacquaniti et al., 1999; Ivanenko et al., 2008) was modeled by simple oscillators coupled with appropriate time shifts (Barliya et al., 2009), indicating that combination of oscillatory commands may be used for movement coordination (Hoellinger et al., 2013). Concerning the sport domain, in the elite fencers but not in amateurs, intensive training of the thrusting movement of the upper limb induces the emergence of a surprising whipping movement (Cheron et al., 2011). The prime mover action for this rapid elbow extension is made, seemingly paradoxically by the biceps muscles (not by the triceps) acting as a biarticular muscle for performing fast elevation of the arm that induces a dynamic interaction torque at the elbow. This emergent functional sport strategy was perfectly predicted by a DRNN receiving the EMG envelop of eight superficial muscles of the upper limb as input signals (Cheron et al., 2011). Different approaches have been developed on the base of artificial recurrent neural networks (RNN) in which different optimization algorithms and different input-output mapping are imposed. For example, in order to increase biological plausibility the Kalman filter was introduced and learned by an RNN which was then able to reproduce the attractor dynamics of cortical circuit (Denève et al., 2007; Linsker, 2008). Recently, Sussillo et al. (2015) demonstrated in the monkey that it was possible to reproduce complex EMG patterns of multiple muscles of the arm during a reaching task by feeding the RNN with seven neuronal signals recorded in the motor and premotor cortex. This elegantly demonstrates that the cortical dynamics is able by itself to produce naturalistic solutions (Sussillo, 2014) expressed in the EMG output patterns. This also corroborates our preliminary results showing that electroencephalographic (EEG) signals used as input to the DRNN were able to reproduce walking movement in human (Cheron et al., 2012).

## From the tensegrity concept to neuronal oscillations

These biomechanical evidences are in line with the new ecological concept of tensional integrity, or tensegrity, a structural principle describing spatial systems made of isolated prestressed components in compression inside a net of continuous tension (Turvey and Fonseca, 2014). As a contraction of tension and integrity, the tensegrity concept (Levin, 2006), combines the different levels of physical links from cellular to tissue and body architectural connectivity. In movement science, tensegrity integrates force distribution into a complex network of passive and active tissues including nerve sensors. This addresses both the production of force and movement with temporary deformation of body tissue. In the absence of external force, the tensegrity configuration assumes the stabilization of the whole body (Skelton and de Oliveira, 2009). Given the richness of tissue innervation (cutaneous, articular and muscular), the tensegrity concept also encompasses another central issue in movement science and sport psychology: haptic perceptual systems. The field of multiple forces acting on the different micro-regions of the tensegrity configuration of the body activates the emergence of a haptic envelope. In practice, the conceptual idea of the haptic cube (Kugler and Turvey, 1987) requires technological adaptation (Bernstein et al., 2013) in order to record stress vectors on the body surface. Such haptic assemblage can also serve the emerging field of intelligent textile (De Rossi et al., 2011; Tormene et al., 2012) in particular for applications dedicated to competitive sport (Rogowski et al., 2006). From a more basic perspective, more knowledge needs to be developed about the neuronal processing of haptic information. For example, the recent study of Jörntell et al. (2014) demonstrated that different haptic features are initially encoded into rich representations in the cuneate neurons in the medulla oblongata which is well before the information reaches the somatosensory cortex. Though this pathway is seen as part of the sensory system, the intricate nature of motor commands and sensory information must be borne in mind. This is well illustrated by reports of astronauts in weightlessness indicating that they can completely lose their limb perception in relaxed condition until a voluntary muscle contraction restore the related limb perception (Clement and Reschke, 2008). The important role of descending information not only from premotor and motor cortex but also from cortical system normally devoted to the treatment of ascending information is now largely recognized. The neural support for the tensegrity concept could be provided by resonance, synchrony and oscillation. For example, solicitation of body tensegrity may concern the alpha brain oscillation (8–12 Hz). This oscillation is viewed as an active inhibitory mechanism (Klimesch, 2012) that gates and controls the cognitive relevance of sensorimotor processing (Sadaghiani et al., 2012). This rhythm is modified in weightlessness (Cheron et al., 2006, 2014) in such a way that phase-locking of theta-alpha oscillations related to the perception of a 3D tunnel image was suppressed in weightlessness. The phase-locking mechanism largely explains why information related to movement overcomes artificial sensory input. For example, when the median nerve is electrically stimulated at the wrist, a negative evoked potential (the N30 component) emerges in the frontal area, mainly (~70%) produced by the phase locking of beta-gamma oscillation. This phase locking is completely suppressed when subjects move the stimulated hand (Cebolla et al., 2009). This promotes the idea that the oscillatory phase-locking may gate the different sensory inputs arising during sport movement depending of the relationship between environment and the body at rest or in action. Another important point which may help to join tensegrity concept and brain oscillations is that brain oscillations are adapted to the timing properties of the mechanics of the effector system including the contraction speed of myosin and actin (Buzsáki et al., 2013).

## Integration of human biological signals into oscillatory approach

Integrated analysis of multiple biological signals while the body is in movement will undoubtedly provide key insights into the psychological determinants of sport performance.

The development of reliable wireless systems greatly facilitates the recording of simultaneous signals recorded during whole body movements, such as EMG, EEG, eye movements, skin sensors. However, the profusion of the recorded data necessitates not only important storage capacity but also strategic management and conceptual guidelines. The fact that all of these biological signals coming from both streams of the sensori-motor loop are by nature oscillatory may promote the emergence of new field in movement science and sport psychology. Following the identification of central pattern generators (CPG) in the brainstem and spinal cord generating rhythmical movements (e.g., breathing, walking or swimming) in the absence of sensory input but “learning CPGs” are also present in the neocortex, where they lead to the emergence of spontaneous dynamics (Yuste et al., 2005).

It is now considered that the information processing by the brain is essentially dynamic rather than static (Tsubo et al., 2013). Churchland et al. (2012) elegantly demonstrated that simple, non-periodic reaching movements are generated by oscillatory patterns of cortical neurons population resembling those that produce rhythmic movements. A theta-gamma oscillation code between motor cortex and hippocampus has been proposed on the basis of local field potentials and neuronal firing recorded during single voluntary movement (sequence of push-hold-pull of a lever) in the rat (Igarashi et al., 2013). Theta oscillation (4–10 Hz) was present during hold period and reduced during the movement. This slow oscillation was accompanied by (1) gamma oscillation (30–50 Hz) during the lever hold period and by (2) fast gamma oscillation (60–120 Hz) starting before the onset of lever pull and ending after the termination of movement. It was also demonstrated that the neuronal firing of the pyramidal cells of the motor cortex are phase-locked to gamma oscillation during the holding period. The development of EEG dynamics tools coupled with non-invasive recording, inverse modeling approach (e.g., swLORETA, to access the functional generators of oscillation) and specific stimulation devices such as transcranial direct current stimulation, and a virtual reality environment would allow a rapid and promising expansion of this oscillatory approach in humans. The study of neuronal rhythm dynamics is thus very important to understand how these rhythms facilitate final operational decisions. Sensory, motor and cognitive processing are associated with specific brain oscillations related to subcortical and neocortical structures. These oscillations are considered to be able to filter incoming signals, to prime the network for plasticity and to tune motor commands. High-density EEG recordings have revealed brain oscillatory signatures of motor actions and imagery (Ramos-Murguialday and Birbaumer, 2015) that are particularly relevant to skilled movement.

For example, prior to a self-paced movement an event related desynchronization (ERD) of the alpha-beta rhythm is recorded over the contralateral sensorimotor cortex followed by a bilateral alpha-beta ERD (Pfurtscheller and Neuper, 2006) and by a contralateral event related synchronization (ERS) after the movement (Pfurtscheller and Lopes da Silva, 1999). In addition to these sensorimotor rhythms, theta (Landau and Fries, 2012), and gamma (Brunet et al., 2015) oscillations also sculpt brain activities in a succession of ERD/ERS sequences encompassing the global dynamics of the nervous system at rest, during observation, imagination and action.

The extreme limits reached by sport competition also offer a privileged domain for deciphering the presence and the modulation of these different neuronal oscillations linked to success or failure but always realized in an optimized state of performance. This may represent one of the future Grand Challenges of Movement Science and Sport Psychology.

For this purpose, it has become experimental approaches of sport movement that link together an array of biological signals have become vital, leading to functional integration of biomechanics (e.g., kinematics, kinetics), physiology (e.g., breathing, blood circulation) including neurophysiology (e.g., EEG, EOG, EMG, neuroimaging), and neuropsychology.

Then, the functional coupling of these experimental data should be modeled by dedicated RNN allowing simulation of movement performance dynamics. The results of this “oscillatory approach” should be transmitted to the trainers of teams to serve as a more solid basis for individualized training. Further along the cycle, this approach should be utilized to evaluate outcomes and design improved training programs. We predict that this Frontiers topic will serve as an important platform for integrating studies dedicated to a better understanding of the neural determinants of sport performance.

## Funding

I would like to thank B. Dan for fruitful discussion about the manuscript, T. D'Angelo M. Dufief, E. Toussaint, E. Hortmanns, and M. Petieau, for expert technical assistance. This work was funded by the Belgian Federal Science Policy Office, the European Space Agency (AO-2004,118), the Belgian National Fund for Scientific Research (FNRS), the research funds of the Université Libre de Bruxelles and of the Université de Mons (Belgium), the FEDER support (BIOFACT), the MINDWALKER project (FP7–2007–2013) supported by the European Commission, the Fonds G. Leibu and the NeuroAtt BIOWIN project supported Walloon Country.

### Conflict of interest statement

The author declares that the research was conducted in the absence of any commercial or financial relationships that could be construed as a potential conflict of interest.
